# Barriers, facilitators and strategies for the implementation of artificial intelligence‐based electrocardiogram interpretation: A mixed‐methods study

**DOI:** 10.1111/eci.14387

**Published:** 2025-04-07

**Authors:** Bauke K. O. Arends, Jenna M. McCormick, Pim van der Harst, Pauline Heus, René van Es

**Affiliations:** ^1^ Department of Cardiology University Medical Center Utrecht Utrecht The Netherlands; ^2^ Graduate School of Life Sciences University Utrecht Utrecht The Netherlands; ^3^ Cordys Analytics B.V Utrecht The Netherlands; ^4^ Cochrane Netherlands University Medical Center Utrecht, Utrecht University Utrecht The Netherlands

**Keywords:** artificial intelligence, Delphi, electrocardiogram, mixed‐methods, pre‐implementation

## Abstract

**Introduction:**

The implementation of artificial intelligence‐based electrocardiogram interpretation (AI‐ECG) algorithms relies heavily on end‐user acceptance and a well‐designed implementation plan. This study aimed to identify the key barriers, facilitators and strategies for the successful adoption of AI‐ECG in clinical practice.

**Methods:**

A sequential explanatory mixed‐methods study was conducted among future AI‐ECG end‐users in the Netherlands, including doctors, nurses, and ambulance professionals, using a clinical scenario involving chest pain. Quantitative data were collected through a three‐round Delphi survey (*n* = 25) to identify key barriers and facilitators. Building on these findings, qualitative data were gathered through semi‐structured interviews (*n* = 7) and focus groups (*n* = 12) to further explain the barriers and facilitators, and discuss relevant implementation strategies.

**Results:**

Participants expressed a general openness to working with AI‐ECG. Four key barriers and twelve facilitators were identified in the quantitative phase. Participants mentioned the relative advantage of AI‐ECG in the context of recognizing subtle, or rare, ECG abnormalities and assisting in patient triage. However, successful implementation requires end‐users to have trust in the algorithm, clear protocols, actionable model output, integration with existing clinical systems and multidisciplinary implementation teams. Several strategies were proposed to address these challenges, including conducting local consensus discussions, identifying and preparing local champions and revising professional roles.

**Conclusions:**

This mixed‐methods study grounded in established theoretical frameworks identified several barriers and facilitators to AI‐ECG implementation and proposed strategies to address these challenges. These findings provide valuable insights for developing effective implementation plans for AI‐ECG in clinical practice.

## Graphical abstract

This study aimed to identify barriers, facilitators and strategies for the implementation of artificial intelligence‐based electrocardiogram interpretation algorithms. We conducted a sequential explanatory mixed‐methods study, including a Delphi survey, semi‐structured interviews and focus groups among future end‐users of these algorithms. We identified key barriers and facilitators relevant for successful implementation, and propose suitable implementation strategies.

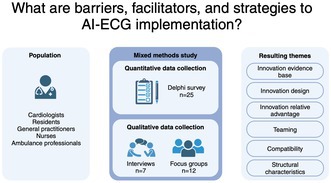

## 1 INTRODUCTION

Artificial intelligence‐based electrocardiogram interpretation (AI‐ECG) is an emerging field undergoing rapid growth, as evidenced by the increasing number of published studies, which now exceed 2500.^1^ Successful applications of AI‐ECG include prediction of occlusion myocardial infarction,^2^, ^3^ left ventricular systolic dysfunction,^4^ and paroxysmal atrial fibrillation.^5^ Despite this surge in research, clinical implementation of these algorithms remains limited. As of 2020, only 40 AI‐driven devices in cardiovascular medicine have received approval from the United States Federal Drug Administration or the European CE mark.^6^, ^7^ This significant gap between research and practical application, often referred to as the ‘valley of death’, is a well‐recognized phenomenon across various fields.^8^, ^9^

Bridging this gap requires the development and application of effective strategies that facilitate the successful integration of AI‐ECG into clinical practice. Implementation science plays an important role in this process by formulating strategies that both address anticipated key barriers and leverage facilitators encountered during the implementation process.^10^ Engaging end‐users early during the development process allows for the technology to be tailored to meet the specific needs of diverse clinical sectors, thereby increasing its chances of successful adoption.^11^ Frameworks such as the Consolidated Framework for Implementation Research (CFIR) and Expert Recommendations for Implementing Change (ERIC) are valuable tools in this regard, systematically assessing barriers and facilitators and developing strategies for implementation within healthcare contexts.^12^, ^13^

Recent studies on the clinical implementation of AI have mainly focused on perspectives from field leaders or physicians,^14^, ^15^, ^16^ although select research engaged a more diverse group of stakeholders.^17^ However, none of these studies specifically addressed the implementation of AI‐ECG. We hypothesize that engaging a diverse group of future AI‐ECG end‐users, including these beyond traditional leadership roles, will yield valuable insights into the relevant barriers and facilitators to clinical implementation of AI‐ECG. Furthermore, these stakeholders are likely to propose practical strategies to overcome these barriers and leverage facilitators, thereby contributing to the successful integration of AI‐ECG algorithms in clinical practice.

## 2 METHODS

### 2.1 Overview

**FIGURE 1:**
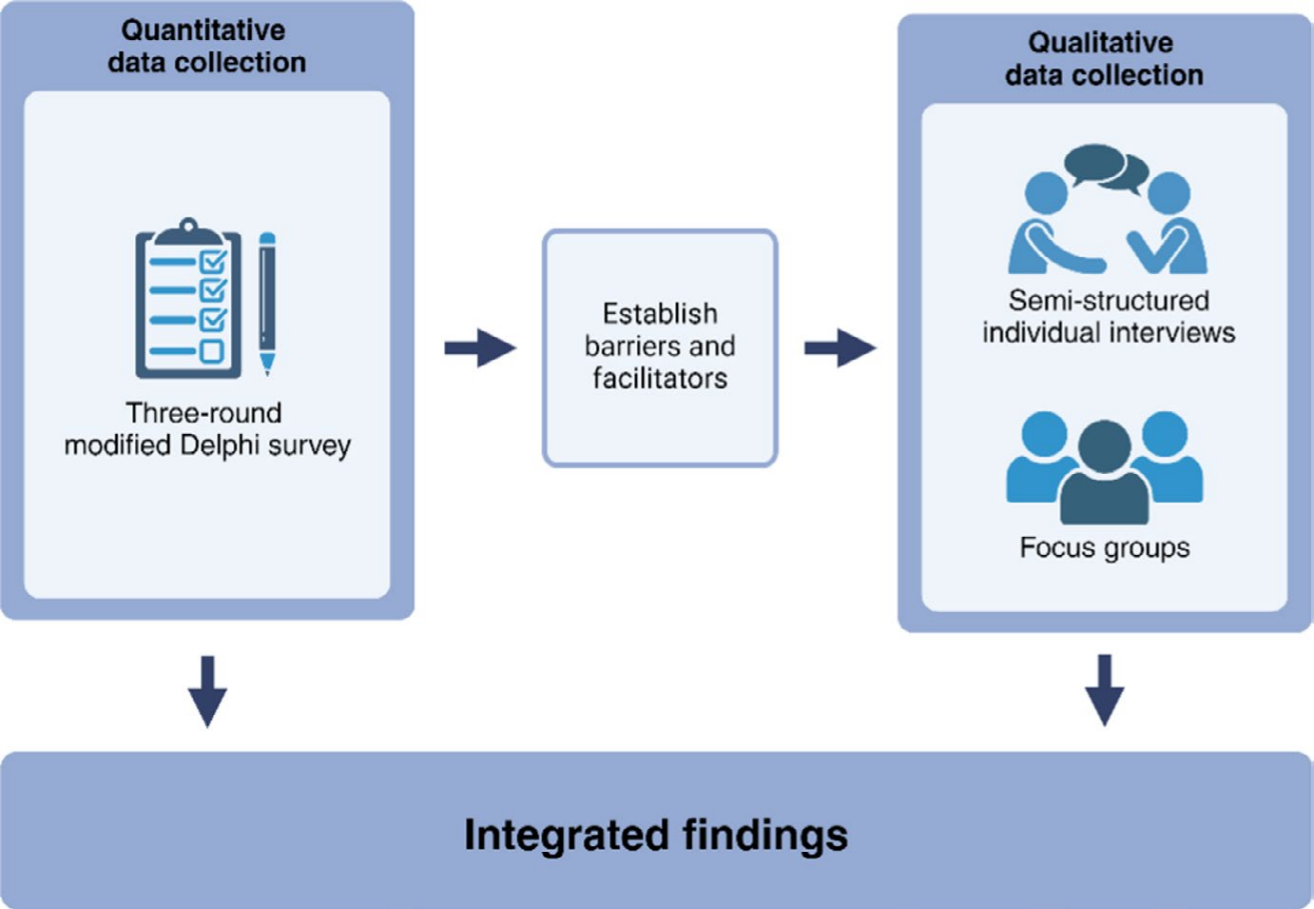
High‐level overview of the mixed‐methods sequential explanatory study design. There is no overlap in participants between the three data collection methods.

This mixed‐methods study employed a sequential explanatory design, wherein quantitative data were first collected and analysed (Delphi survey), followed by qualitative data (interviews and focus groups) to provide further explanation and context (Figure 1). Participants were potential end‐users of AI‐ECG, including cardiologists, residents, general practitioners (GPs), nurses, ambulance professionals and support staff. Recruitment, data collection and analysis were conducted between May 2023 and July 2024. Participants were recruited through various channels, including online social media platforms, posters, the author's professional networks, public email lists, and snowball sampling. Each participant participated in only one of the following: the Delphi survey, semi‐structured interview or focus groups.

This study was exempt from institutional ethics review as it did not fall under the Medical Research involving Human Subjects Act (WMO). All participants provided written informed consent prior to participation, and all data were anonymized before analysis. This study adhered to the COREQ reporting guidelines (Appendix S1).^18^

### 2.2 Quantitative data collection

We conducted a three‐round modified electronic Delphi survey to identify barriers and facilitators to the implementation of AI‐ECG algorithms. Before committing to participation, potential participants were provided with a comprehensive summary of the study, detailing the research topic, methodology and timeline. Participants were requested to maintain anonymity throughout the survey process and to refrain from discussing their responses with others. Open recruitment for participants during the first round lasted until response saturation was reached. Response saturation, also referred to as data saturation, is a concept in qualitative research that indicates the point at which no new themes, insights or information are emerging from additional data collection. During the second and third rounds, weekly survey reminders were sent to participants who had completed the preceding round.

The first survey round primarily involved gathering background information and brainstorming. The survey was designed in an hourglass format, beginning with general questions about the use of AI in clinical practice and gradually narrowing down to a scenario involving an AI‐ECG algorithm for patients with chest pain. At the time of data collection, such an algorithm was under development by our research group. This scenario was included to make the concept of AI‐ECG more concrete and to stimulate more focused responses. AI‐ECG was defined as any deep learning algorithm that uses the ECG as input.

Participants' responses were consolidated into two comprehensive lists of barriers and facilitators. Two authors (BA and JM) independently analysed the initial responses, followed by discussions to reach inter‐coder agreement. One researcher employed a deductive coding approach after a literature review of existing barriers and facilitators on AI implementation in healthcare, while the other used an open and axial inductive coding approach. Each barrier and facilitator was assigned a unique identifier, code and description and was mapped to a corresponding CFIR construct and domain.

The second round of the survey aimed to achieve consensus. Participants were presented with all identified established barriers and facilitators and asked to indicate in a yes/no format whether they agreed that each item was relevant to the implementation of an AI‐ECG algorithm. Consensus was defined as ≥70% agreement among all participants. An optional text box was provided for participants to elaborate on their responses. Additionally, participants could suggest any additional barriers and facilitators that were not yet listed. The same two authors reviewed these suggestions, but none warranted the addition of new items.

The final round involved ranking all remaining barriers and facilitators in order of importance using a relative numerical ranking, with a lower number indicating greater importance. An optional text box was provided for comments and justification.

### 2.3 Qualitative data collection

To complement the Delphi survey responses, we conducted individual semi‐structured interviews and focus groups. The primary objective of these qualitative methods was to gain a deeper understanding of the identified barriers and facilitators, as well as to explore strategies for overcoming the barriers and leveraging the facilitators. Participants for the qualitative phase were recruited from the same population and through the same methods used for the Delphi survey. Interviews and focus groups were conducted either in person at the participants place of work or via Microsoft Teams, according to participant preference. All sessions were recorded using Microsoft Teams, regardless of whether they were face‐to‐face or online. Automatically generated transcripts were reviewed and corrected in a non‐verbatim manner. Transcripts were not returned to participants for comment or correction.

### 2.4 Semi‐structured individual interviews

All interviews were conducted by JM, a female master's student without any prior interview experience or previous relationship with the interviewees. Interviews were conducted in English and lasted approximately 30 minutes. A topic list was designed to begin with general questions about AI in healthcare and narrow to address specific scenarios as well as barriers and facilitators previously identified in the questionnaire (Appendix S2). Our aim was to include participants until data saturation was reached.

### 2.5 Focus groups

Two focus groups, each lasting approximately one hour, were conducted by BA, a male physician with prior interview experience but no prior relationship to the interviewees. The focus groups were conducted in Dutch, with one group consisting of physicians and the other of nurses. A topic list was developed to explore specific issues that emerged during the semi‐structured individual interviews (Appendix S3).

### 2.6 Overall analysis and integration

Quantitative results were analysed using descriptive statistics and are presented as numbers and percentages or as means and standard deviations. Mean rankings and standard deviations were calculated for all barriers and facilitators, with lower scores indicating that the participant perceived this barrier or facilitator as more important to implementation.

Qualitative data were manually coded using NVivo version 1.7. Implementation strategies were deductively coded based on the ERIC framework. Two researchers (BA and JM) independently coded the interviews and focus groups, followed by discussions to establish inter‐coder agreement. After coding, we conducted a framework analysis based on the CFIR to integrate the quantitative and qualitative findings and extract common themes. This integrated analysis allowed us to provide a comprehensive understanding of the barriers, facilitators and strategies for the implementation of AI‐ECG algorithms.

## 3 RESULTS

Tables 1 and 2 display the demographic characteristics of all participants. Forty participants started the Delphi survey, with twenty‐five participants completing all rounds, leading to a drop‐out of 38%. Semi‐structured interviews were held among seven participants, and two focus group sessions were conducted, one including six nurses and one including six physicians. Data saturation was reached after these interviews and focus groups, as no new concepts emerged. Due to both direct and indirect recruitment methods, the response rate for the Delphi survey is unknown. The response rate for the interviews and focus groups is estimated at 70%.

**TABLE 1:** Demographic characteristics survey participants.

	*N* (%)^a^
Age, years	
Mean (range)	42 (27–61)
Work experience, years	
Mean (range)	11 (1–33)
Profession	
Cardiologist	5 (20)
Resident cardiology	2 (8)
GP	2 (8)
Nurse	2 (8)
Ambulance professional	8 (32)
ECG technician	4 (16)
IT technician	1 (4)
Other medical specialists	1 (4)
Gender	
Female	5 (20)
Male	20 (80)
Review ECGs	24 (96)
Have used a rule‐based computer algorithm for ECG analysis or triage	14 (56)
Have used AI‐ECG in clinical practice	1 (4)
Are open to using AI‐ECG	25 (100)
AI algorithms are used in clinical practice within their field	13 (52)
Believe AI‐ECG could improve the accuracy and speed of ECG analysis and triage for patients with chest pain	23 (92)
Are concerned about the potential for errors of AI‐ECG in chest pain patients	15 (60)
Believe healthcare professionals should be trained to effectively use AI‐ECG for patients with chest pain	19 (76)
Believe ethical or social considerations should be addressed when implementing AI‐ECG for patients with chest pain	18 (72)
Believe patients will have a positive response to the implementation of AI‐ECG	17 (68)

^a^ Participants who completed all three rounds.

**TABLE 2:** Demographic characteristics qualitative data collection.

Identifier^a^	Profession	Age	Sex	Work experience (years)
I1	Nurse	26	Female	3
I2	Cardiologist	35	Male	2
I3	Resident cardiology	30	Female	2
I4	Cardiologist	56	Male	18
I5	Cardiologist	64	Male	25
I6	General practitioner	39	Male	9
I7	Professor	37	Female	8
FN1	Nurse	37	Female	12
FN2	Nurse	28	Female	5
FN3	Nurse	35	Female	10
FN4	Nurse	64	Female	44
FN5	Nurse	26	Female	5
FN6	Nurse	56	Female	38
FD1	Resident cardiology	34	Male	6
FD2	Resident cardiology	36	Male	6
FD3	Cardiologist	40	Male	7
FD4	Cardiologist	49	Female	15
FD5	General practitioner	34	Female	3
FD6	General practitioner	46	Male	17

Abbreviations: FD, focus group doctors; FN, focus group nurses; I, interview.

^a^ Participant dropout during the rounds of the Delphi survey. During each round, all available data was used, so the number of participants, and the resulting demographic characteristics, differ between the first and third round.

In both the quantitative and qualitative phase, participants indicated that they were not currently working with AI‐ECG in clinical care. In contrast, almost all participants indicated to be open to working with this technology. Four barriers and twelve facilitators identified in the Delphi survey, including rankings and associated CFIR constructs, are detailed in Table 3. Out of the four barriers, ‘Poor model performance’ held the highest importance (mean rank 1.5 ± 0.9), while ‘Faster recognition of subtle ECG abnormalities’ (mean rank 4.1 ± 1.6) was the most important facilitator. The CFIR construct ‘Innovation’ encompassed the majority of barriers and facilitators,^11^ with ‘Inner setting’ accounting for three, and ‘Implementation process’ and ‘Outer setting’ each accounting for one.

**TABLE 3:** Barriers and facilitators and corresponding CFIR constructs and domains.

	CFIR domain	CFIR construct	Rank
			Mean (SD)
Barrier			
Poor model performance	Innovation	Innovation evidence base	1.5 ± 0.9
Missing ECG quality check	Innovation	Innovation relative advantage	2.6 ± 1.1
Lack of integration with existing workflows	Inner setting	Compatibility	2.9 ± 0.9
Poor presentation of model output	Innovation	Innovation design	3.0 ± 0.9
Facilitator			
Faster recognition of subtle ECG abnormalities	Innovation	Innovation relative advantage	4.1 ± 2.8
Clinical benefit	Innovation	Innovation relative advantage	4.4 ± 2.8
Triage in the pre‐hospital setting	Innovation	Innovation relative advantage	4.8 ± 2.9
Improved triage and resource allocation	Innovation	Innovation relative advantage	5.0 ± 3.3
Recognizing rare ECG abnormalities	Innovation	Innovation relative advantage	6.1 ± 3.5
End‐user training	Inner setting	Access to knowledge and information	6.3 ± 3.0
Detailed protocols	Inner setting	Structural characteristics – Work infrastructure	6.4 ± 3.4
Triage in the hospital setting	Innovation	Innovation relative advantage	7.1 ± 3.0
Algorithm explainability	Innovation	Innovation design	7.9 ± 2.5
Financial benefit	Innovation	Innovation cost	8.4 ± 3.0
Involvement of key participants	Implementation process	Teaming	8.7 ± 2.8
Legal considerations	Outer setting	Policies & laws	8.9 ± 3.3

Abbreviations: CFIR, Consolidated Framework for Implementation Research; SD, standard deviation.

### 3.1 Innovation evidence base

Confidence and trust in the innovation's evidence base emerged as an important theme influencing the implementation of an AI‐ECG algorithm. This concern was reflected in the quantitative data, where ‘poor model performance’ was identified as the most important barrier. In interviews and focus groups, stakeholders frequently expressed concerns about relying on flawed AI outputs, fearing that inaccurate results could lead to adverse outcomes, increased diagnostic testing or incorrect treatment plans.

The worst [scenario] would be that you relied on false algorithm output that indicated […] don't send this patient to the emergency room, […], and the patient dies because of the mistake. I6

To address these concerns, participants suggested strategies such as the development and distribution of educational materials, holding educational meetings, developing and implementing tools for quality monitoring and conducting local consensus discussions. However, the effectiveness of these strategies depends on the availability of high‐quality evidence demonstrating the model's performance. Without such evidence, even the best educational efforts will not fully alleviate scepticism. Despite these initiatives, some participants remained wary. When comparing AI‐ECG algorithms to non‐AI medical devices, participants noted that the complexity and opacity of AI algorithms required additional efforts to build confidence and trust. One participant noted that it would help most if the use of specific algorithms was implemented in clinical practice guidelines.

If it [the AI‐ECG algorithm] is listed in a guideline, then I do have confidence in that. I can't check everything [the evidence base] individually. FD6

### 3.2 Innovation design (innovation domain)

Explainability of AI‐ECG algorithms emerged as a topic of mixed responses among participants. Some participants believed that having explainability would add significant value, enabling clinicians to better understand the rationale behind AI‐generated outputs and thus creating trust in the technology. However, others disagreed, arguing that explainability might be less essential than the overall accuracy and reliability of the model's predictions. Additionally, the presentation of the model's output was emphasized as a critical factor. Participants highlighted the importance of outputs being directly actionable. To address this concern, participants suggested strategies including facilitate relay of clinical data to providers, tailor strategies and assess for readiness and identify barriers and facilitators.

Moreover, participants described the importance of algorithm adaptability to different populations and clinical settings. For example, they suggested that cardiologists might require a more detailed output compared to GPs. Beyond the presentation of the output, participants also mentioned the need for setting‐specific thresholds within the algorithm, allowing for different approaches, such as rule‐in or rule‐out, depending on the clinical context. A potential strategy to address this is tailor strategies.

### 3.3 Innovation relative advantage and cost (innovation domain)

Participants frequently compared AI‐ECG to current clinical practice, generally viewing it as a facilitator, which aligned with several facilitators identified in the quantitative study component. The main advantages highlighted were the faster recognition of subtle and rare ECG abnormalities, particularly in settings where the expertise in ECG interpretation is not readily available. These capabilities were seen as a potential improvement to patient triage in prehospital settings and non‐cardiology wards. However, within the cardiology department, participants felt that the algorithm offered little relative advantage.

Nearly all participants viewed AI‐ECG as an additional diagnostic tool or data point to inform clinical judgement, rather than as a replacement for it. The most common concern expressed was that AI‐ECG is unable to incorporate the complete clinical picture.

Ideally, […] any AI recommendation is only part of the complete clinical evaluation and then the clinician ultimately decides what to do. I6

Financial considerations also emerged as a potential factor influencing the implementation of AI‐ECG. While financial benefit was listed as a facilitator in the quantitative data, it was only sparsely mentioned during qualitative discussions. One general practitioner noted the importance of a reimbursement plan to ensure uptake. However, financial aspects were not a prominent theme among participants, suggesting that cost considerations may play a secondary role in their perceptions of AI‐ECG implementation.

### 3.4 Teaming (implementation process domain)

Participants felt that an ideal implementation team should contain both medical experts and technical experts, along with individuals who possess knowledge in both fields. One focus group participant highlighted the importance of this interdisciplinary approach:

Crucial to this point [implementation teams] is people who can do a little bit of both. […] Someone who can bridge that [medical and technical expertise], is essential when bringing the innovation to the end‐user. FD1

Additionally, another participant noted that knowing the individuals on the implementation team can help facilitate trust in the algorithm:

With AI, I need to have a basic trust in the people who are working on it. […] That's why I like having people in our own department who I know and have worked with for a long time. I trust them, and I trust their group. I5

However, some participants argued that the evidence base is more important for establishing trust in the algorithm than any explanation or presentation provided by the implementation team:

You're more interested in the performance of the test in different populations. It doesn't matter how it's developed, as long as it has good diagnostic value. FD2

Related strategies that were discussed include identify and prepare champions, create new clinical teams and use data experts.

### 3.5 Compatibility (inner setting domain)

Participants across various clinical settings indicated the importance of ensuring compatibility when integrating an AI algorithm into existing workflows and IT systems. For GPs, the current process of conducting an ECG and consulting with a cardiologist involves multiple steps, such as scanning, emailing and waiting for an interpretation. This process highlights the need for seamless integration into existing EHR systems to streamline these tasks. Cardiologists and residents also noted the necessity of viewing current and previous ECGs simultaneously to track changes over time. Therefore, successful implementation of AI‐ECG would require its compatibility with GP and hospital EHR systems, as well as ambulance monitoring devices. Strategies to resolve this include promote network weaving, change physical structure and equipment and use data warehousing techniques.

### 3.6 Structural characteristics (inner setting domain)

The need for detailed protocols emerged as a key theme, underscoring the importance of role delineation before implementing AI‐ECG algorithms into clinical practice. This sentiment was expressed by nurses in the focus group, who emphasized the need for clear guidelines on how to act on specific AI‐generated outcomes.

This concern was echoed by a GP in the physicians focus group, who highlighted the necessity for care network agreements to ensure consistency across different levels of care. The GP stressed the importance of having standardized protocols, specifically mentioning the fear that a medical specialist might deny a referral because they do not consider the AI algorithm's output as a valid referral criterion:

What I don't want is to call [a cardiologist] and have them respond: ‘The algorithm may say that, but I don't think the ECG is abnormal’. FD6

Strategies to assist in overcoming these issues include revise professional roles and promote network weaving.

## 4 DISCUSSION

This mixed‐methods study aimed to identify key barriers, facilitators and strategies for the implementation of AI‐ECG algorithms among end‐users. Although these users are not currently working with AI‐ECG in clinical practice, they expressed openness to adopting such technologies in the future. Participants recognized the potential value of AI‐ECG algorithms in enhancing current clinical practices, such as faster and more accurate recognition of ECG abnormalities and improved patient triage. However, to realize this potential, trust and confidence in the algorithms must be established. Additionally, the model must produce actionable output and be integrated into existing clinical workflows. Successful implementation should involve an interdisciplinary team comprising both medical and technical experts, with clear protocols established prior to deployment. The most commonly mentioned strategies for implementation include conducting educational meetings, holding consensus discussions, revising professional roles and tailoring strategies.

This study is one of few that focus specifically on the practical implementation of AI algorithms in the cardiovascular field. Another relevant study explored stakeholder perspectives on implementing an AI‐based clinical decision support tool aimed at reducing readmission risk for heart failure patients.^19^ Several themes overlap between the two studies, including concerns about trust in the algorithm and the issue of ‘technology fatigue’, which parallels our findings on the need for integration into clinical workflows and existing IT systems.

In our study, the identified barriers and facilitators were categorized within the CFIR domains of the inner setting, innovation, and implementation process, while the outer setting and individuals domains were less represented. This pattern aligns with findings from a review on barriers and facilitators of AI implementation in healthcare, which also noted a stronger focus on these specific domains.^20^ Unlike some studies which pre‐selected relevant CFIR constructs, we chose to remain open to including any CFIR domain or construct, allowing for a more comprehensive exploration of factors affecting implementation.^21^

The uneven representation of CFIR domains in our findings may reflect the fact that end‐users are less concerned with or less frequently interact with the outer setting and individual domains. This could be because these domains are often managed at an organizational or administrative level, rather than by the end‐users themselves. Therefore, including policymakers or managers may have led to different results.

Although financial benefit was listed as a facilitator in our study, it was not frequently mentioned by end‐users during qualitative data collection. This is in line with previous work among physicians, but contrasts with findings from interviews with field leaders in machine learning, who may be more exposed to financial aspects of AI implementation.^14^, ^15^, ^21^ The payment model of the healthcare system may also play a role, and in the Netherlands, where healthcare costs are tightly regulated and shared between insurers and the government, financial aspects may not be as immediate a concern for clinicians.

In this study, participants expressed differing viewpoints on the need for AI‐ECG explainability. Some viewed it as essential, while others were less convinced of its importance. This divergence reflects the broader discourse in current research, where the topic of explainable AI continues to be widely debated.^19^, ^22^, ^23^, ^24^, ^25^, ^26^ These differing opinions complicate predictions about the impact of explainable AI on end‐user acceptance during implementation. Further studies and discussions are needed to reach a consensus on the role and form that explainability should take in the context of AI‐ECG.

This study has several limitations that should be considered when interpreting the findings. Some stem from the inherent nature of qualitative research, while others are tied to the specific methods employed in this study. First, although only one clinical scenario was referenced, the results we found were comparable to other work on implementation of AI in healthcare. Combined with the broad range of end‐users we interviewed, this suggests that our findings may be generalizable beyond the context studied. However, incorporating additional clinical scenarios or extending the study to other countries could provide further insights into the generalizability and applicability of the findings. Second, the AI‐ECG algorithm discussed is not yet a fully developed product. The absence of a concrete, finalized innovation may have impacted participants' perceptions of its relative advantage, potentially leading to the omission of certain CFIR constructs that might have been more relevant with a finalized product, leading to superficial or incomplete findings. Additionally, this study may be subject to self‐selection bias, as the participants who chose to take part in this study may have had a more positive or receptive view of AI‐ECG compared to those who did not participate. This bias could have led to a limited diversity in viewpoints and an overestimation of the readiness or enthusiasm for adopting AI‐ECG among the broader population of end‐users.

Another limitation concerns the scope of the qualitative data collection. Ambulance professionals were not included due to the difficulty in recruiting willing participants. As a result, insights from this group, which has a unique prehospital perspective, are only drawn from the quantitative data collection, which may lead to an incomplete understanding of all challenges and needs associated with AI‐ECG implementation in this setting. Furthermore, the limited sample size of our study may restrict the representativeness of the findings, as it may not fully capture the diversity of experiences and perspectives among potential end‐users, and the fact that all participants were from the Netherlands limits the generalizability of our findings to the whole of Europe, or beyond. Finally, the responses gathered were exclusively from the perspective of end‐users. While their insights are essential, the successful implementation of AI‐ECG will require input from a broader range of stakeholders, including device manufacturers, patients, managers, and policymakers. These additional perspectives are needed for addressing practical, regulatory, and ethical considerations that may not have been fully explored in this study.

## 5 CONCLUSION

We identified potential key barriers and facilitators to the implementation of AI‐ECG from the perspective of end‐users and explore implementation strategies to address these challenges. Successful implementation of AI‐ECG requires addressing concerns related to model performance, ensure integration with clinical workflows and algorithm adaptability to specific clinical settings, form an interdisciplinary implementation team, and develop detailed clinical protocols prior to deployment. These insights can guide AI‐ECG developers and implementation teams in creating a robust and effective implementation plan for the adoption of AI‐ECG in clinical setting.

## AUTHOR CONTRIBUTIONS

Bauke Arends: conceptualization, investigation formal analysis and writing—original draft preparation. Jenna McCormick: conceptualization, investigation, formal analysis, writing—reviewing and editing. Pim van der Harst: conceptualization, writing—reviewing and editing, supervision. Pauline Heus: methodology, writing—reviewing and editing. René van Es: conceptualization, writing—reviewing and editing and supervision.

## CONFLICT OF INTEREST STATEMENT

RvE is cofounder, shareholder and board member of Cordys Analytics B.V., a spin‐off of the UMC Utrecht that has licensed several AI‐ECG algorithms. The UMC Utrecht receives royalties from Cordys Analytics for potential future revenues.

## Supporting information


Appendix S1.

Appendix S2.

Appendix S3.

## Data Availability

Data used in this study are not publicly available due to privacy concerns.
