# Supplementary material for: Barriers, facilitators and strategies for the implementation of artificial intelligence‐based electrocardiogram interpretation: A mixed‐methods study
Source: Eur J Clin Invest. 2025 Apr 7;55(Suppl 1):e14387. doi: 10.1111/eci.14387 (PMC11973840; doi:10.1111/eci.14387)
Supplement: Supplementary file 2 — Appendix S2. [file ECI-55-e14387-s002.pdf]

Semi-structured interview guide

Intro

- “I would like to thank you for taking the time to meet with me today. The research we are working on aims to determine consensus on the clinical implementation of an AI-algorithm that functions to analyze and triage ECGs. The research involves electronic questionnaires, one on one interviews, and focus groups. The electronic questionnaires aim to reach consensus among participants on the identification of barriers and facilitators for implementation of the algorithm. The objective of the one on one interviews is to address how to overcome the barriers and utilize the facilitators for the algorithm’s implementation. As a [profession], your clinical expertise and opinion is highly valued for our research.
- The interview should take approximately 30 minutes. I will be audio recording the interview for analysis purposes. The recording will be kept confidential, within our research team, and any responses included in the final report will not be linked back to you as a respondent.
- Are there any questions about everything I have explained so far?
- If not, please sign the informed consent document and we will begin.”

- ****Obtain informed consent, sign PIF****
- ****Begin audio recording of Teams meeting****

- Define AI & provide examples
- **The algorithm under development in the UMC Utrecht**
  - The algorithm under development is capable of automatically analyzing ECGs of patients with chest pain and providing an assessment. The algorithm uses only the ECG and no other patient information. The outcome categorizes a patient as normal, abnormal, or acute.

Background info

- Name: ___________________________________
- Profession: _______________________________
- Age: _________
- Work experience (years): ____________

Questions

- **Intro questions (AI in general)**
  - AI current role & experience
    1. Please describe the current role of AI in your clinical field.
       a. Is it used at all?
       b. Is it viewed as a positive innovation? Why or why not?
    2. How do you feel about the extent of AI use in your clinical field at present?
       a. Should it be used less/more?
       b. Why do you feel that way?

3. Could you explain your personal experience with AI-technologies and ECG analysis tools?
    a. Do you believe your colleagues have the same experience?
    b. If no, what makes your experience different?

- **Barriers and facilitators**
  Briefly introduce the survey results. Our survey yielded several barriers and facilitators related to the implementation of AI, which we would like to discuss in more detail.

- **Focus questions**
  - Facilitators & barriers for implementation
    4. Which factors would facilitate working with an AI-algorithm?
        a. How could these factors be implemented?
    5. Which factors would make it difficult to work with an AI-algorithm?
        a. What would be needed to overcome these difficulties?
    6. What strategies can be employed to gain acceptance and buy-in from all members of the healthcare team, including doctors, nurses, and administrative staff?
  - Future perspectives & recommendations
    7. How do you envision the future of this AI-algorithm in your area of clinical practice? What improvements or updates might be necessary down the line?
    8. What would be a worst case scenario when using an AI model in your clinical practice?
    9. What would be an ideal situation when using an AI model in your clinical practice?
    10. What would be the most important factor for sustainable implementation of AI in healthcare?
  - AI-algorithm performance
    11. How can we measure the AI-algorithm's impact on patient outcomes and overall efficiency of our healthcare system?
  - Risk mitigation
    - **Context** - In the event of algorithm implementation, there is a risk that clinicians will rely solely on the algorithm's output and in turn experience a decrease in their own personal knowledge and skills.
    12. Are there any potential risks associated with over-reliance on the AI-algorithm, and how can we prevent or mitigate them?
    13. How can we strike a balance between relying on the AI-algorithm's suggestions and maintaining the expertise of our clinicians?
    14. In cases where the AI-algorithm's results conflict with a clinician's assessment, how should the final decision be made? What should be the hierarchy of decision-making? Should this differ depending on the end user?

- Legal & ethical considerations
  15. Are there any legal or ethical considerations related to implementing this AI-algorithm? How can we ensure patient data privacy and consent?

<u>Outro</u>

- **Closing remarks**
  16. "Is there anything more you would like to add?
  17. I will be analyzing all of the interviews, with the goal of developing implementation strategies for the AI-algorithm. If you are interested in the final analysis of all interviews, I can send you a copy upon completion.
      a. Yes / no
  18. Thank you for your time. Your insights are crucial to understanding the technical logistics that must be addressed for successful implementation of an AI-algorithm into clinical practice."

****End & save audio recording of Teams meeting****
